# The Association between Leisure and Physical Activity Level with Depressive Symptoms after 5-years of follow-up

**DOI:** 10.1093/geroni/igab046.2891

**Published:** 2021-12-17

**Authors:** Milan Chang, Hrafnhildur Eymundsdottir, Alfons Ramel, Sigurveig Sigurdardottir, Vilmundur Gudnasson, Lenore Launer, Palmi Jonsson

**Affiliations:** 1 The Icelandic Gerontological Research Institute, Reykjavik, Hofuoborgarsvaoio, Iceland; 2 Landspitali University Hospitali, Landspitali University Hospitali, Hofuoborgarsvaoio, Iceland; 3 University of Iceland, Reykjavik, Hofuoborgarsvaoio, Iceland; 4 National Institute on Aging, Bethesda, Maryland, United States; 5 University hospital Landspitali, Reykjavik, Hofuoborgarsvaoio, Iceland

## Abstract

BACKGROUND: Depressive symptoms in older adults are associated with socioeconomic status (SES), medical care, and physical activity. However, there is little evidence on the longitudinal association between level of leisure activity (LA) and physical activity (PA) with depressive symptoms among community-dwelling older adults in Iceland. The study examined an association of LA and PA at baseline with high depressive symptoms (HGDS) assessed after 5 years of follow-up among community-dwelling older adults. METHODS: A large community-based population residing in Reykjavik, Iceland participated in a longitudinal study with 5 years of follow-up (n=2957, 58% women, 74.9±4.8 yrs). Those with HGDS or dementia at baseline were excluded from the analysis. The reported activity was categorized into 2 groups as no-activity versus any-activity. Depressive symptoms were assessed by the 15-item Geriatric Depression Scale (GDS) on average 5 years later. RESULTS: After adjusting for demographic and health-related risk factors, those who reported having any LA had significantly fewer HGDS after the follow-up of 5 years (6 or higher GDS scores, Odds Ratio (OR) = 0.46, 95% Confidence Interval (CI): 0.27 ~ 0.76, P = 0.003). However, reporting any PA at baseline was not significantly associated with HGDS (OR = 0.71, 95% CI: 0.51 ~ 1.00, P = 0.053). CONCLUSION: Our study shows that any LA among older adults is associated with having less depressive symptoms 5 years later among community-dwelling older adults while having any PA was not associated with depressive symptoms after 5 years of follow-up.

